# Depressive symptoms, pain and disability for adolescent patients with juvenile idiopathic arthritis: results from the Childhood Arthritis Prospective Study

**DOI:** 10.1093/rheumatology/key088

**Published:** 2018-04-25

**Authors:** Laura Hanns, Lis Cordingley, James Galloway, Sam Norton, Livia A Carvalho, Deborah Christie, Debajit Sen, Roberto Carrasco, Amir Rashid, Helen Foster, Eileen Baildam, Alice Chieng, Joyce Davidson, Lucy R Wedderburn, Kimme Hyrich, Wendy Thomson, Yiannis Ioannou

**Affiliations:** 1 Arthritis Research UK Centre for Adolescent Rheumatology, University College London, London, UK; 2 Arthritis Research UK Centre for Epidemiology, University of Manchester, Manchester, UK; 3 Department of Academic Rheumatology, Faculty of Life Sciences & Medicine, London, UK; 4 Psychology Department, Institute of Psychiatry Psychology and Neuroscience, Kings College London, London, UK; 5 Department of Clinical Pharmacology, William Harvey Research Institute, Queen Mary University of London, London, UK; 6 Child and Adolescent Psychological Services, University College London Hospital NHS Foundation Trust, London, UK; 7 Paediatric Rheumatology, Institute Cellular Medicine, Newcastle University, Newcastle upon Tyne, UK; 8 Department of Rheumatology, Alder Hey Children’s Foundation NHS Trust Liverpool, Liverpool, UK; 9 Department of Rheumatology, Royal Manchester Children’s Hospital, Manchester, UK; 10 Department of Rheumatology, Royal Hospital for Children, Glasgow, UK; 11 Paediatric Rheumatology, Royal Hospital for Sick Children, Edinburgh, UK; 12 UCL GOS Institute of Child Health, University College London, London, UK

**Keywords:** adolescent rheumatology, juvenile idiopathic arthritis, depression, pain, disability

## Abstract

**Objectives:**

To determine if depressive symptoms assessed near diagnosis associate with future measures of pain, disability and disease for adolescent patients diagnosed with JIA.

**Methods:**

Data were analysed from JIA patients aged 11–16 years recruited to the Childhood Arthritis Prospective Study, a UK-based inception cohort of childhood-onset arthritis. Depressive symptoms (using the Mood and Feelings Questionnaire; MFQ), active and limited joint count, disability score (Childhood Health Assessment Questionnaire), pain visual analogue scale and patient’s general evaluation visual analogue scale were collected. Associations between baseline measures (first visit to paediatric rheumatologist) were analysed using multiple linear regression. Linear mixed-effect models for change in the clinical measures of disease over 48 months were estimated including MFQ as an explanatory variable.

**Results:**

Data from 102 patients were analysed. At baseline, median (IQR) age was 13.2 years (11.9–14.2 years) and 14.7% scored over the MFQ cut-off for major depressive disorder. At baseline, depressive symptoms significantly associated with all clinical measures of disease (*P* ⩽ 0.01). High baseline depressive symptoms scores predicted worse pain (*P* ⩽ 0.005) and disability (*P* ⩽ 0.001) 12 months later but not active and limited joint counts.

**Conclusions:**

Adolescent patients with JIA and depressive symptoms had more active joints, pain and disability at the time of their first specialist appointment. The associations between baseline depression and both pain and disability continued for at least one year, however, this was not the case for active joint count.

**Rheumatology key messages**

- Depressive symptoms are highly prevalent in adolescents with JIA in the UK.
- Depressive symptoms predict future disability and pain but not active disease for adolescent JIA patients.
- A psychological intervention study aiming to improve long-term outcomes for adolescent JIA patients is warranted.

## Introduction

Adolescence is recognized by the WHO as ‘the period in human growth and development that occurs after childhood and before adulthood, from ages 10 to 19’ [1]. Adolescence is a time of immense biological, social and vocational change [2]. It is also a high risk period for the development of mental health problems with approximately half of all psychiatric disorders starting between late adolescence and early adulthood [2]. For these reasons, adolescence is considered an important and yet vulnerable period of development that is distinct from both childhood and adulthood.

JIA is defined as arthritis of unknown aetiology that begins before the age of 16 years and persists for a minimum of 6 weeks [3]. It comprises seven distinct categories of heterogeneous conditions as defined by the ILAR [3]. The prevalence of JIA in the UK is approximately 1 in 1000 children [4].

Adolescents with rheumatic disease experience substantial functional limitations in leisure, home and school activities [5]. The causes are most likely multifactorial revolving around pain, disability, fatigue, disease unpredictability and illness perception [5–7]. It is therefore unsurprising that many studies have found that young people with JIA are at increased risk of experiencing depression compared with their healthy peers [8–11]. Furthermore, the JIA categories that commonly have an age of onset during adolescence (polyarthritis, extended oligoarthritis and enthesitis-related arthritis) tend to run a more severe course and are the least likely of the subtypes to go into treatment-free remission [12]. This makes it especially pertinent to determine the burden of low mood in this age group with JIA and to explore the relationships between mood and clinical measures of disease.

For adolescent patients with JIA, the relationship between mood and clinical measures of disease remains poorly defined. This is partially due to the difficulty of unpicking all of the underlying mechanisms as well as the limitations of the few existing studies that included both children and adolescents with varying disease duration and age of onset [13, 14]. To date, no studies have undertaken a longitudinal analysis of these associations in a purely adolescent JIA inception cohort.

This study explores the hypothesis that for adolescents with JIA, depressive symptoms are associated with clinical measures of disease (active and limited joint count, disability, pain and the patient’s general evaluation of disease) at the patient’s first visit to a paediatric rheumatologist and that baseline depressive symptoms may predict future clinical outcomes.

## Methods

Data for this study came from JIA patients recruited to the Childhood Arthritis Prospective Study (CAPS), a UK based inception cohort of childhood onset arthritis. Details of the study have been described elsewhere [15]. Ethical approval to undertake the study was given by the UK Northwest Multicentre Ethics Committee (No. 01/8/104). Written informed consent from parents/guardians of all patients was obtained and all able children provided informed assent. Consent was obtained according to the Declaration of Helsinki (updated 2008). Patients were recruited within 6 months of their first visit to a paediatric rheumatologist. Data collected at the first study visit were labelled baseline data. Patients were followed up longitudinally at 6 months, annually to 5 years and then at 7 and 10 years.

### Clinical and psychological assessments

Depressive symptoms were assessed at baseline using the long version of the child self-report Mood and Feelings Questionnaire (MFQ) (Angold A, Costello EJ, Pickles A, Winder F., unpublished work) with a score of 0 indicating no depressive symptoms and a score of 66 indicating very high depressive symptoms. A score of ⩾27 was used as a cut-off score for major depressive disorder (MDD). This cut-off score has been shown to give the best diagnostic confidence with good sensitivity and specificity [16]. The continuous score version of the MFQ (0–66 score) was used in all other analyses.

Clinical measures of disease were recorded; number of joints considered clinically active or restricted in motion by the rheumatologist (active and limited joint counts), disability (Child HAQ), pain (0–10 cm visual analogue scale; VAS) and the patient’s general evaluation of disease (PGE: 0–10 cm VAS).

**Fig. 1:**
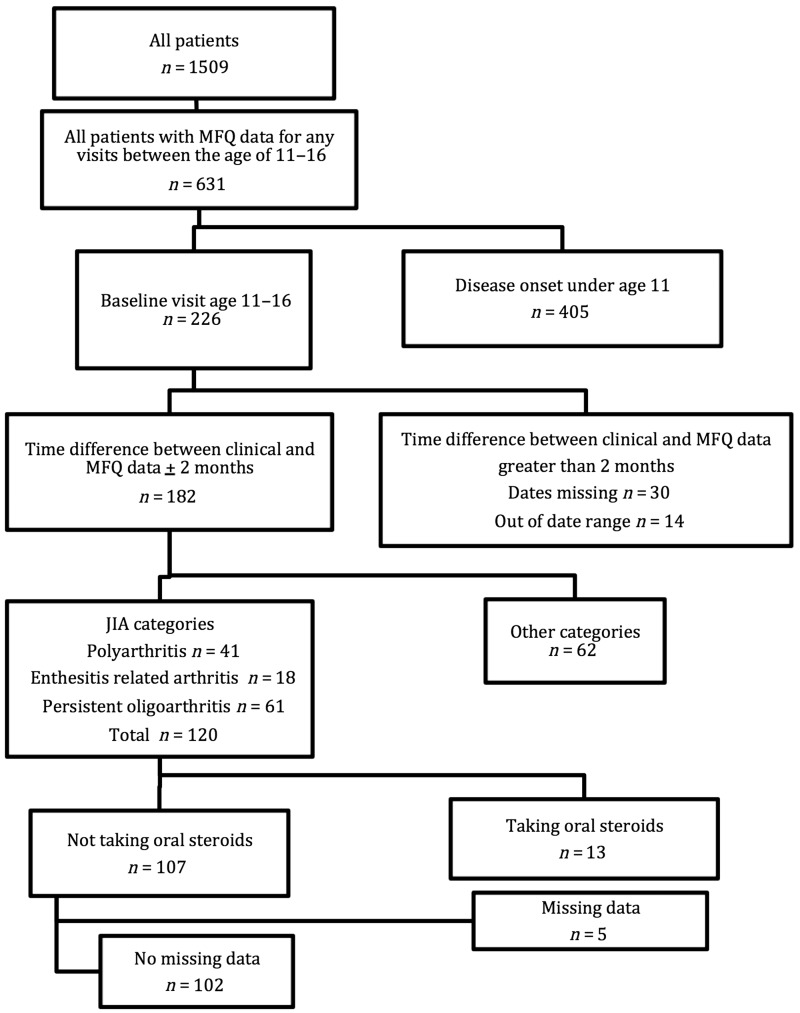
Inclusion criteria Patients were included in the data analysis if they met all criteria shown in the flow chart. Failure to meet any of these criteria resulted in exclusion. Polyarthritis refers to both RF positive and negative polyarthritis. No missing data refers to the following variables at baseline: age, gender, JIA category, MFQ score, DMARD use, MFQ score, active and limited joint counts, disability, pain and the patient’s general evaluation of disease. CHAQ: Child Health Assessment Questionnaire; MFQ: mood and feelings questionnaire; PGA: patient’s general evaluation; VAS: visual analogue scale.

The anticipated effect size of mood on disease activity was small. It was therefore important to limit the amount of variation in disease activity that could be attributed to other variables. Due to limitations in sample size, not all variables could be appropriately controlled for in the regression analysis and so a number of inclusion criteria were applied. Patients selected for inclusion in this analysis were aged 11–16 years at baseline, had completed the MFQ within 2 months of their baseline assessment, had an ILAR JIA diagnosis of polyarthritis (RF positive and negative), oligoarthritis or enthesitis-related arthritis and had complete data at baseline for all variables investigated (Fig. 1). There were too few patients in the systemic arthritis, PsA and undifferentiated arthritis categories for analysis to be appropriately powered, and so these patients were excluded from further data analyses. Patients were excluded if taking oral steroids at baseline as the effect of oral steroids may have masked any associations between mood and worsened disease activity.

### Cross sectional analysis of baseline data

Data were tested for normal distribution by the Kolmogorov–Smirnov test. Data were not normally distributed and so non-parametric tests were used. Associations between clinical measures of disease, age, gender, JIA category and depressive symptoms at baseline were analysed using Spearman’s correlation and the Mann–Whitney test.

Five separate multiple linear regression models were performed looking at the association between clinical measures of disease and depressive symptoms at baseline. Each model included a different clinical measure of disease as the dependent variable. Five separate analyses were performed due to high collinearity between the different clinical measures of disease. Depressive symptoms (continuous score) were included as the independent variable. Age, gender and use of DMARDs were controlled for in all models regardless of significance in Spearman’s correlation and the Mann–Whitney tests. Additionally, for the disability, pain and PGE models, JIA category was controlled for. JIA category was not controlled for in the active and limited joint count models due to high collinearity. Standardized and unstandardized beta values are presented.

### Longitudinal analysis

Linear mixed effects models for change in clinical measures of disease and depressive symptoms over 48 months were estimated. This model implicitly imputes missing data based on trends in the rest of the study population data for that variable. As a result, no patients were lost over time due to missing data. Time was included in the model as a linear spline with a change point at 12 months, which allowed for different rates of change between 0–12 months and 12–48 months. Later time points were not included due to limited data. Baseline depressive symptoms were then included in the models of disease outcome as an explanatory variable. This model was then used to predict clinical measures of disease over time for a representative low (2 points) and high (31 points) depressive symptoms score at baseline. This longitudinal analysis was not powered to account for other covariates due to the small sample size. To test the difference between the estimated clinical measures of disease at 12 months for the representative high and low baseline depressive symptoms scores, a *Z* test for differences in estimated means was calculated. Data were analysed using SPSS Statistics version 21 (IBM Corp., Armonk, NY, USA) and STATA/IC version 12 (StataCorp LLC, College Station, TX, USA).

## Results

### Patient selection

A total of 102 patients were included in the analysis. Table 1 shows the baseline demographic and clinical information for the 102 patients included in the analyses. No patients had been treated with a biologic at baseline. At baseline, 14.7% of patients scored over the cut-off score of 27 for MDD (Table 1).

**Table 1:** Baseline socio-demographic and clinical data

Variable	
All patients, *n* (%)	102 (100)
Gender, *n* (%)	
Female	58 (56.9)
Male	44 (43.1)
Age (11–16 years), median (IQR), years	13.2 (11.9–14.2)
Time between first visit to a paediatric rheumatologist and baseline study visit, median (IQR), months	0.8 (0.3–1.8)
JIA category, *n* (%)	
Polyarthritis (RF positive and negative)	31 (30.4)
Oligoarthritis	53 (52.0)
Enthesitis-related arthritis	18 (17.6)
DMARD at baseline, *n* (%)	
Yes	6 (5.9)
No	96 (94.1)
Depressive symptoms, MFQ score (0–66), median (IQR)	13.0 (6.0–20.0)
Patients over cut-off for depressive symptoms, MFQ score 27+, *n* (%)	15 (14.7)
Active joint count (0–72), median (IQR)	2.0 (1.0–5.0)
Limited joint count (0–72), median (IQR)	1.0 (1.0–4.0)
Disability, CHAQ (0–3), median (IQR)	0.625 (0.000–1.125)
Pain, 0–10 cm VAS, median (IQR)	3.2 (0.9–6.0)
PGE, 0–10 cm VAS, median (IQR)	2.5 (0.7–4.9)

One hundred and two patients meeting the inclusion criteria were included in subsequent analyses. CHAQ: Child Health Assessment Questionnaire; MFQ: mood and feelings questionnaire; PGA: patient’s general evaluation; VAS: visual analogue scale.

### Depressive symptoms associate with clinical measures of disease at baseline

Relationships between depressive symptoms and measures of disease outcomes at baseline were first investigated in a cross-sectional analysis. At baseline in the correlation analysis, depressive symptoms correlated with active joint count (*r* = 0.324, *P* < 0.001), limited joint count (*r* = 0.356, *P* < 0.001), disability (*r* = 0.514, *P* < 0.001), pain (*r* = 0.394, *P* < 0.001) and PGE (*r* = 0.431, *P* < 0.001). Females had significantly higher depressive symptoms [median (interquartile range), 15.5 (7.0–21.0)] than males [10.0 (4.0–15.5); *U* = 967.0, *P* < 0.05]. Depressive symptoms were significantly different between JIA categories at baseline. Patients with polyarthritis had significantly higher depressive symptoms [19.0 (14.0–33.0)] than patients with oligoarthritis [10.0 (4.0–16.0); *U* = 427.5, *P* < 0.001] and enthesitis-related arthritis [9.5 (8.0–16.0); *U* = 156.0, *P* < 0.05]. There were no significant differences in depressive symptoms between patients with oligoarthritis and enthesitis-related arthritis.

The regression analyses showed depressive symptoms at baseline to be associated with active and limited joint counts, disability, pain and PGE after accounting for possible confounding variables (Table 2). The unstandardized beta coefficient represents how many units the dependent variable (clinical measures of disease) changes in relation to a 1-unit increase in the independent variable (depressive symptoms). As different clinical measures of disease utilize different units, it is not possible to compare the strength of the association with MFQ across these measures with the unstandardized beta values. The standardized beta coefficient shows how many standard deviations the dependent variable changes for a 1 s.d. increase in the independent variable. Therefore, the standardized beta scores allow comparison of the strength of the different associations across different clinical measures of disease.

**Table 2:** Baseline associations between different disease outcomes and depressive symptoms

Dependent variable	MFQ unstandardized beta value	*P*-value	95% CI unstandardized beta	MFQ standardized beta value
Active joint count	0.196	0.005	0.062, 0.269	0.330
Limited joint count	0.166	0.006	0.050, 0.258	0.283
Disability (CHAQ)	0.022	<0.001	0.012, 0.377	0.032
Pain (0–10 cm VAS)	0.072	<0.001	0.029, 0.323	0.116
PGE (0–10 cm VAS)	0.082	<0.001	0.043, 0.390	0.12

At diagnosis, depressive symptoms are significantly associated with active and limited joint count, disability, pain and PGE after accounting for covariates. Data were analysed using multiple linear regression models. Age, gender and DMARD use were controlled for. Additionally, for the disability, pain and PGE regression models, JIA category [polyarthritis (RF positive and negative), oligoarthritis and enthesitis-related arthritis] was additionally controlled for. JIA category was not included in the active and limited joint count models due to high collinearity. *n* = 102 adolescent patients with JIA. CHAQ: Child Health Assessment Questionnaire; MFQ: mood and feelings questionnaire; PGA: patient’s general evaluation; VAS: visual analogue scale.

### Depressive symptoms at baseline predict disability, pain and PGE at 4 years, but not active and limited joint counts

**Fig. 2:**
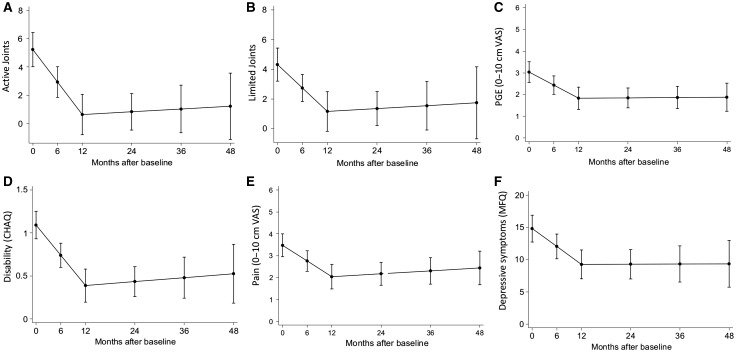
Change in clinical measures of disease and depressive symptoms over 48 months Linear mixed effects models are shown for change in clinical measures of disease and depressive symptoms over 48 months for adolescent patients with JIA. Disease activity measures explored were active joint count (**A**), limited joint count (**B**), PGE (**C**), disability (**D**), pain (**E**) and depressive symptoms (**F**). 95% CI is shown. CHAQ: Child Health Assessment Questionnaire; MFQ: mood and feelings questionnaire; PGA: patient’s general evaluation; VAS: visual analogue scale.

Next, we explored whether depressive symptoms at baseline could predict future clinical measures of disease. Longitudinal analysis showed the estimated change in clinical measures of disease and depressive symptoms over the first 4 years (Fig. 2). Depressive symptoms, active joint counts, limited joint counts, disability, pain and PGE all showed a similar profile: improvement during the first year and then stabilization (Fig. 2).

**Fig. 3:**
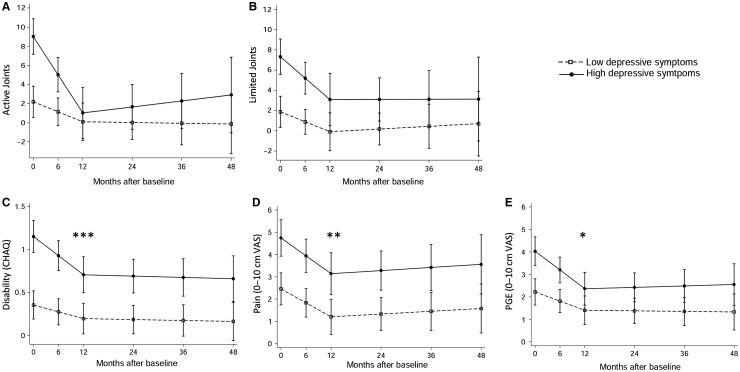
Clinical measures of disease over 48 months for high and low depressive symptoms at baseline Linear mixed effects models are shown for change in clinical measures of disease over 48 months for adolescent patients with JIA: active joint count (**A**), limited joint count (**B**), disability (CHAQ; **C**), pain (**D**) and PGE (**E**). MFQ at diagnosis included in all models. Estimated disease activity for high (31 points, solid line) and low (2 points, dashed line) depressive symptoms shown with 95% CI. *Z* test for difference between estimated scores; *P < 0.05, **P < 0.01, ***P < 0.001. CHAQ: Child Health Assessment Questionnaire; MFQ: mood and feelings questionnaire; PGA: patient’s general evaluation; VAS: visual analogue scale.

Depressive symptoms at baseline were then included in the models of clinical measures of disease over time. These models were then used to predict clinical measures of disease over time for a representative low (2 points) and high (31 points) depressive symptoms score at baseline (Fig. 3). For all outcomes, levels improved between baseline and 12 months and then remained stable up to 4 years. At 12 months, the estimated active joint count and limited joint count for those with high and low depressive symptoms at baseline were not significantly different (Fig. 3). In contrast, the estimated disability and pain scores at 12 months were significantly worse for those with high baseline depressive symptoms (Fig. 3). Similar differences were also found at 48 months. For PGE at 12 months, there was a significant difference in estimated PGE scores between those with high and low baseline depressive symptoms. However, the two predicted scores had overlapping confidence intervals (Fig. 3). The estimated clinical measures of disease at 12 months, 95% CIs and *Z*-score values can also be found in supplementary Table S1, available at *Rheumatology* online.

## Discussion

The aim of this study was to investigate the associations between depressive symptoms and disease activity for adolescent patients with JIA at their first visit to a paediatric rheumatologist and longitudinally over the following 4 years. At baseline 14.7% of patients had significant depressive symptoms. A higher number of depressive symptoms at baseline associated with worsened clinical measures of disease at baseline. Baseline depression predicted worse pain and disability for up to 4 years later, but did not predict active or limited joint counts.

There were a significant number of young people with JIA who had low mood at baseline; 14.7% of patients scored over the cut-off score for MDD. There have been few studies investigating the prevalence of depression in JIA, particularly during adolescence. Of the few studies that do exist, prevalence rates reported are between 7 and 23% [5, 8, 11]. The prevalence of diagnosed depression for healthy 13- to 15-year-olds in the UK is 1.9% [17]. However, no population norms are available for the MFQ, making it difficult to directly compare our results with depression rates in adolescents without long-term physical health conditions. There have been mixed reports as to whether JIA patients have higher depressive symptoms than healthy children [13, 18]. However, existing studies mostly investigated a mixed population of children and adolescents and use different measures of depressive symptoms and so cannot be directly compared with our findings. At baseline, depressive symptoms were higher for females than for males. This gender difference is well known. From the age of 13 years, females have an increased risk of developing depression, and this risk increases throughout adolescence [19].

Methotrexate is known to cause nausea and vomiting in a high proportion of adolescent patients with JIA [20], which may impact negatively on mood and quality of life. However, as only six patients in the present study were taking DMARDS at baseline, a comparison of depressive symptoms between those taking and not taking DMARDs at baseline could not be made but does warrant future investigation for adolescents with JIA. There was no association between DMARD use at 12 months and depressive symptoms at 12 months.

Longitudinal analysis showed that all clinical measures rapidly improved over the first year and then stabilized. Since patients were recruited to this study within 6 months of their first visit to a paediatric rheumatologist, in the first year of this study many patients would have started taking DMARDs and biologics, begun physiotherapy and may have adopted new self-management behaviours. All of these factors may have contributed to the reduced disease activity scores during the first year of this study. It is interesting that depressive symptoms also improved over the first year and then stabilized, rather than continuing to improve over the following years when disease activity was generally low. This pattern of depressive symptoms over time mirrors the pattern of disease activity over time. There are four possible explanations for this mirrored pattern of symptomology; depressive symptoms may lead to worsened disease activity, disease activity may lead to depressive symptoms, an external factor common to both depressive symptoms and disease activity may lead to this pattern of symptoms over time, or a combination of all of the above factors. Further studies are required to test these hypotheses and determine the directionality of these relationships. The results from our cross sectional regression analysis and longitudinal analysis showed that for adolescent JIA patients, baseline depressive symptoms were associated with worsened pain and disability at baseline and this relationship persisted over the next 4 years. For many patients, this may mean worse pain and disability as they enter adulthood. What few studies there are into the relationship between mood, pain and disability for patients with JIA largely support the findings of this current study. Depressive symptoms have previously been shown to associate concurrently with disability and pain for children and adolescents with JIA [13, 18, 21]. Longitudinally, depressive symptoms have been shown to predict future pain and disability, but only when initial levels of pain and disability are low [14]. In order to identify effective treatment targets it will be important to investigate the inflammatory, cognitive and behavioural mechanisms that may explain these relationships in a purely adolescent JIA population.

For adult RA patients, many studies have shown that depressive symptoms strongly associate with both current and future pain and disability [22–26]. However, despite there being more published research in adult RA patients, the mechanisms behind mood, pain and disability still remain unclear for these patients.

Depression and pain share common biological pathways. The decreased noradrenaline and serotonin associated with depression affect neurotransmission in pain pathways, resulting in reduced pain inhibition and a worse experience of pain [27]. Furthermore, adolescents who use catastrophizing as a coping strategy for pain also report more depressive symptoms, which may be due to the rumination type behaviours common to both [28]. This is particularly relevant for adolescents. The prefrontal cortex (responsible for self-control) does not fully mature until adulthood whereas the limbic system (responsible for emotional reactivity) is already fully developed [28]. This results in a bias towards poor cognitive emotion regulation, which in turn associates with depressive symptoms [29, 30]. The pain experienced by JIA patients can lead to reduced physical activity, particularly if the patient believes that exercising will cause further joint damage, disability and pain [31, 32]. This decreased activity may lead to a cycle of physical deconditioning, worsened disability and worsened pain [31, 33]. In addition, the social withdrawal associated with depression is likely to contribute to reduced physical activity and exacerbate this cycle [31]. It is important to acknowledge the effect of worse disability and pain on lifestyle limitations, quality of life and therefore mood.

We found that baseline depressive symptoms associated with higher PGE scores at baseline. However, the longitudinal results are less clear. At 12 months, the estimated PGE scores were significantly higher with high baseline depressive symptoms. However, the significance level was approaching 5% and CIs were overlapping, and so a firm conclusion is difficult to make. It may be that the sample is underpowered to detect a difference or that no true association exists. A previous study looking at children and adolescents with JIA found that higher depressive symptoms associated with higher concurrent PGE scores [18]. No studies that we are aware of have previously explored this relationship longitudinally.

Our cross-sectional analysis showed that baseline depressive symptoms also associated with a higher active joint count and limited joint count at baseline. Two studies have previously explored the association between active joint count and depressive symptoms for JIA patients in a cross-sectional analysis but neither study found an association [13, 18], which contradicts our findings. The reason for this discrepancy is unclear but may be due to differences in study design and age of patients included. Both previous studies investigated a mixed population of both children and adolescents with varying disease duration (between 5 months and 15 years), had lower sample sizes, used different measures of depressive symptoms and did not account for any covariates in their analysis [13, 18]. Neither study investigated limited joint count.

Our longitudinal analysis showed that after the first year baseline depressive symptoms no longer associated with subsequent active joint count or limited joint count. We are not aware of any previous studies exploring the longitudinal association between depressive symptoms and these clinical measures of disease in adolescents with JIA. In adult RA patients, depressive symptoms have been shown to associate with a higher active joint count at the same point in time [34] and with a higher tender joint count, physician VAS and a composite DAS28 2 years later [25]. Other studies have found no associations [24, 26]. It is important to bear in mind that these studies were in an adult RA population and so cannot be directly compared with the results from our analysis in adolescent JIA patients.

The association between active inflammatory disease and depressive symptoms may partially be explained by the inflammatory theory of depression, which states that depression can be driven by peripheral inflammation [35]. This means that patients with chronic inflammatory diseases, such as JIA, may have an increased susceptibility to developing depression. This theory is supported by proof of concept studies in adults [36–38], children [39] and mouse models [40]. Depressive symptoms have been shown to associate with changes in glucocorticoid sensitivity [25, 41], increased oxidative stress [42, 43], changes in lymphocyte populations [44, 45] and inflammasome activation [46]. One may hypothesize that these changes may contribute in some adolescent patients with JIA towards lowering the threshold for flares. However, it is important to acknowledge that the directionality behind the association between inflammation and depression remains unclear and is likely to be multifactorial and bi-directional.

This study has some limitations. Though the MFQ is a widely used and accepted measure of depression with excellent reliability and validity [16, 47], it was originally designed to screen for severe depressive symptoms in psychiatric patients and so is not so sensitive to lower levels of depressive symptoms. While it has been useful to identify patients over the cut-off score for MDD in this population, the MFQ may not be sensitive enough in detecting or measuring low-level depressive symptoms. The longitudinal analysis was not powered to include age, gender, medication use and JIA category as confounders due to study dropouts over time.

This study has shown for the first time that depressive symptoms are highly prevalent in adolescents with JIA in the UK. This finding justifies actively screening for depressive symptoms in routine clinical practice. We have also shown that depressive symptoms associate with worse pain, disability and active disease. Crucially, depressive symptoms at baseline predict future disability and pain but not active disease. This work highlights the need for a psychological intervention study with the aim of improving long-term outcomes for adolescents with JIA.

## Supplementary Material

Supplementary Data
