# Supplementary material for: Depressive symptoms, pain and disability for adolescent patients with juvenile idiopathic arthritis: results from the Childhood Arthritis Prospective Study
Source: Rheumatology (Oxford). 2018 Apr 25;57(8):1381–9. doi: 10.1093/rheumatology/key088 (PMC6055569; doi:10.1093/rheumatology/key088)
Supplement: Supplementary Data [file key088_suppl_data.docx]

**SUPPLEMENTARY DATA**

**Supplementary table S1. Clinical measures of disease at 12 months for a representative baseline low and high depressive symptoms score**

| Dependent Variable | Baseline depressive symptoms | Predicted disease outcome | SE | 95% confidence intervals | | Difference | |
| --- | --- | --- | --- | --- | --- | --- | --- |
|  |  |  |  |  |  | Z score | P value |
| Active joint count | Low | 0.094 | 1.004 | -1.874 | 2.062 | -0.48 | 0.628 |
|  | High | 1.023 | 1.364 | -1.651 | 3.678 |  |  |
| Limited joint count | Low | -0.092 | 0.953 | -1.959 | 1.776 | -1.72 | 0.085 |
|  | High | 3.087 | 1.318 | 0.503 | 5.671 |  |  |
| Disability (CHAQ) | Low | 0.194 | 0.091 | 0.016 | 0.373 | -3.31 | ≤0.001 |
|  | High | 0.706 | 0.107 | 0.496 | 0.916 |  |  |
| Pain  (0-10cm VAS) | Low | 1.206 | 0.405 | 0.412 | 1.999 | -2.79 | ≤0.005 |
|  | High | 3.143 | 0.481 | 2.201 | 4.086 |  |  |
| PGE (0-10cm VAS) | Low | 1.271 | 0.370 | 0.547 | 1.996 | -1.99 | <0.05 |
|  | High | 2.536 | 0.439 | 1.676 | 3.396 |  |  |

The estimated clinical measures of disease at 12 months for a representative low (2 points) and high (31 points) depressive symptoms score are shown along with standard error (SE) and 95% confidence intervals. The z score and p value for the difference between the predicted score for those with high and low baseline depressive symptoms is shown. Linear mixed effects models for change in clinical measures of disease (active joint count, limited joint count, pain, disability and PGE) over 48 months for adolescent patients were generated. A separate model was generated for each clinical measure of disease. Depressive symptoms (MFQ) at diagnosis were included in all models as an explanatory variable. VAS: visual analogue scale; MFQ: mood and feelings questionnaire; CHAQ: Child Health Assessment Questionnaire; PGA: patient’s general evaluation.
